# When, where and who? Accessing health facility delivery care from the perspective of women and men in Tanzania: a qualitative study

**DOI:** 10.1186/s12913-018-3357-6

**Published:** 2018-07-18

**Authors:** Thecla W. Kohi, Lilian T. Mselle, Justine Dol, Megan Aston

**Affiliations:** 1 School of Nursing, Muhimbili University of Health and Allied Sciences, Dar es Salaam, Tanzania; 2 Faculty of Health, Dalhousie University, Halifax, Canada; 3 Dalhousie University School of Nursing, Halifax, Canada

**Keywords:** Material services, Birth care, Humanizing birth, Qualitative, Tanzania, Health facilities

## Abstract

**Background:**

Childbirth is a momentous event for women and their partners, yet women continue to die in childbirth worldwide, particularly in sub-Saharan Africa. To reduce maternal mortality and increase the number of women delivering at health facilities, it is important to understand reasons why women who do deliver at health facilities chose to do so. Therefore, the objective of this qualitative study was to explore the perceptions of women and men on (i) when women go to the hospital; (ii) where women deliver; and (iii) who is involved in the delivery process related to accessing health facilities for delivery care in Tanzania.

**Methods:**

Using a qualitative design, four focus group discussions (*n* = 23) and semi-structured interviewers (*n* = 12) were held with postnatal women and men who were attending a postnatal clinic in the Lake Zone region of Tanzania. Data was analyzed using thematic coding.

**Results:**

Women and men expressed factors that influenced when, where, and with whom they accessed health facilities for delivery care, with the quality of care received providing a significant influence. When decisions were made about going to the hospital, there were challenges that resulted in delayed treatment seeking; however, couples recognized the need to seek care earlier to prevent complications. Private hospitals were the preferred location for delivery with public hospitals and home deliveries with traditional birth attendants being less desirable. Both when and where delivery took place was influenced by the desire for better quality of care received as well as financial costs. Finally, there was mixed evidence on who was involved in decision making around delivery location from the perspective of women and men, but both groups expressed a preference for more male involvement during the delivery.

**Conclusion:**

Men and women show desire for women to delivery at health facilities; however, improvements are needed with respect to maternal care and humanizing the birth process in Tanzania. Greater emphasis needs to be placed on including men during the birth process, improving the quality of care received in public hospitals, and reducing the barriers to accessing health facilities for delivery care.

## Background

In every country, pregnancy and childbirth are momentous events in the lives of women and their partners [1]. Each year, 303,000 women continue to die in childbirth worldwide with the largest portion of death occurring in sub-Saharan Africa, where 201,000 maternal deaths occurred in 2015 alone [2]. Tanzania was ranked 6th among 13 countries with the highest levels of maternal mortality [3]. While the number of Tanzanian women using a health facility for delivery increased from 50% in 2010 to 63% in 2015, this varies depending on whether women live in urban (86%) or rural (54%) location [4]. Only 40% of women delivering in the Lake Zone will access a health care facility compared to 94% in Dar es Salaam [4]. Correspondingly, the perinatal mortality, consisting of stillbirth and early newborn mortality within 7 days, in Tanzania remain high at 39 deaths per 1000 live births [4]. Increasing the number of women having a skilled birth attending at birth and delivering at a health facility is critical to decrease both perinatal and maternal mortality [5].

To increase the number of women delivering at health centers, it is important to understand reasons why women who do deliver at health facilities choose to do so. Exploring reasons women seek delivery at health facilities for preventative care builds on the original three-delay model identified by Thaddeus and Maine to understand reasons for seeking emergency maternal care related to the decision to seek care, late arrival at a health facility, and provision of adequate care [5, 6]. Studies have identified some factors that are associated with the use of maternal facility services including distance to health facility, discussion with male partner on place of delivery, advice from a health care provider to deliver at a health facility during an antenatal care clinic, and knowledge of pregnancy risk factors [5–7]. Other studies have identified health system factors that prohibit women from seeking birth care in health facilities [8], which can be addressing through improving the quality of care received and improving access to respectful maternal care.

Since 2000, there has been an increasing emphasis on humanizing the childbirth process, which occurs when women are provided care in a way that respects their rights as human beings and provides sufficient and adequate care throughout the whole birth process, involving them as active agents and capable of making decisions [9–11]. The need for respectful maternal care was recognized as an essential component of the recently updated recommendations for intrapartum care and safe childbirth by the World Health Organization [12]. For this to occur, women have to make it to health facilities for delivery yet contextual factors around when women go to the hospital, where women deliver, and who in the family makes these decisions impacts a women’s delivery experience. Men in low-resource countries often hold significant power and influence over a woman’s health decision, including their access to and experience of maternal health services [13]. To humanize the birth process in Tanzania, it is essential to further understand how decisions are made about accessing delivery services from both the perspectives of women, men and midwives, particularly as there have been limited studies on how men and women differ or converge in their perspectives in accessing health care facilities for delivery care in Tanzania [14]. Therefore, the aim of the present study is to explore reasons why women who do deliver at health facilities chose to do so.

## Objective

The objective of this qualitative study was to explore the perspectives of women and men on (i) when women go the hospital; (ii) where women deliver; and (iii) who is involved in the delivery process related to accessing health facilities for delivery care in Tanzania.

## Methods

### Study design & setting

This study is part of a larger project exploring community and midwives’ perceptions and practices on humanizing birth care in Tanzania. Using a qualitative design, multiple focus group discussions (FGDs) and semi-structured interviews (SSI) were held with women who had recently given birth and men who were attending a postnatal clinic in the Mwanza and Mara regions in Lake Zone, Tanzania. A woman and man could participate on their own, regardless if their partner participated. Focus groups were used in order to obtain women and men feelings, perceptions and opinions about humanizing birth care while the SSI provided in-depth information on the perceptions and opinions of the groups. The SSI also allowed interviewees to feel more comfortable having a conversation alone with an interviewer and thus feel free to provide their understanding on the issues of humanizing birth care from the community, family and health facilities that might not have been captured in the FGDs.

Lake Zone was chosen as it is one of the regions of Tanzania with the highest maternal mortality rates, with Mara having a maternal mortality ratio of 362 per 100,000 births and 305 per 100,000 births in Mwanza according to the 2012 census [15]. Convenient sampling of women and men were recruited from the postnatal ward based on eligibility criteria until saturation of data was reached where no new information was gathered from adding additional participants. Men and women were required to speak Kiswahili fluently, either have a wife who had recently given birth or to have had two or more spontaneous vaginal/normal deliveries, respectively. To capture the typical birth experience for women, women who have delivered twice or more vaginally were included as they were considered to have more experience with humanizing birth care than primigravida’s while women who undergo caesarian section are taken as abnormal cases.

### Informants and data collection

To explore the perceptions and experiences of humanizing birth care in Tanzania, FGDs and SSIs were held following a standard interview guide related to respectful maternal care. Semi-structured interviews were held separately with women and men which were conducted by fluent Kiswahili speakers and who were also registered nurses. Additionally, a total of four FGDs were held – two with women and two with men. Among women, there was a total of 19 participants, thirteen in two FGDs and six individual SSIs. Women’ age ranged from 20 to 45 years and had at least primary education level, mainly housewives. Among men, there was a total of 16 participants, ten in two FGDs and six individual semi-structured interviews. Men’ age ranged from 25 to 60 years. Most Men had at least a primary education level, worked as motorbike cyclists, and peasants.

Focus group discussions and SSIs were conducted in Kiswahili. All interviews were audio-recorded with permission from participants. Focus group discussions and SSIs were held at convenient places within the hospital and the big shelter trees outside the clinics as identified by participants where privacy was ensured. Each SSI and FGD took approximately 45 min.

### Data analysis

Semi-structured interviews and FDGs were transcribed verbatim into Kiswahili, and then translated into English by hired research assistants fluent in both languages. Descriptive statistics were used to summarize participants’ demographics. Qualitative data was analyzed using thematic coding using the English transcripts. Initial codes were collected and reviewed, duplicates reviewed, and similar codes grouped together [16, 17]. Codes and corresponding quotes were reviewed and re-labeled if necessary [18]. Once all codes were identified, codes were grouped under themes of when, where, and who. The SSI and FGDs yielded rich data whereby no additional themes emerged. This demonstrates that there was sufficient data to develop themes [18].

### Ethical consideration

Ethical approvals were obtained from the National Institute of Medical Research and Muhimbili University of Health and Allied Sciences in Tanzania prior to data collection. Participants were informed about the study orally and in writing and completed a written consent form prior to the interviews commencing, followed by demographics. Participants were informed that their interviews would be recorded and agreed for their de-identified quotes to be used.

## Results

Four themes emerged from this study as shown below in Table 1.

**Table 1:** Themes

SN	Theme
1	When decisions are made about going to the hospital
2	Preference for hospitals over traditional birth attendants for delivery
3	Who is involved in decision making around delivery location
4	Who attends the delivery

Themes arose around when, where and who were going to the hospital with women. Decisions around when women were going to the hospital were influenced due to access to transportation and not wanting to wait a long time, yet also recognized the need to arrive early enough to receive proper care. In terms of where women are delivering, there was preference to deliver at a health facility, particularly private hospitals which provided higher quality of care, but due to cost and location, women delivered at public hospital or with a traditional birth attendant (TBA). In terms of who was influential in where women delivered, men lacked consensus about who was involved in decision making whereas the women expressed that they led the decision making around delivery location. While at the hospital, men and women noted a lack of inclusion of men and other family members during delivery but expressed a desired for greater involvement of men in the delivery process, if men felt comfortable doing so.

### Theme 1: When decisions are made about going to the hospital

Both men and women articulated that decisions were being made individually and together around when they access health facilities for birthing care and were influenced by external factors. For instance, both men and women experienced challenges in getting to the hospital once labour has started due to issues around accessing transportation. Some participants comments that because of the distance to the hospital from their homes, women occasionally delivered while on their way.

The transportation services from villages to hospital centers have been a dominant problem in our communities. Sometimes a woman might delay anticipating labor pain, [and then] it occurs suddenly in relation to transportation challenges. The about-to-deliver mother might delay reaching the hospital hence increasing the magnitude of the problem. (FGD, Man 2)

Challenges around getting to the hospital was related to delays in seeking treatment, with women expressing a desire to wait until they were almost ready to give birth before going to the hospital. While women comment on waiting to seek care at a health facility for her delivery to shorten the amount of time they spent at the hospital, women also recognized the need to access services earlier, so that they would not develop complications.

We go to the hospital early because we care about our wellbeing and we are afraid of complications that could arise out of delivering from home. You never know if you’ll deliver safely or if you’ll encounter complications, so it’s better to go the hospital early. (FGD, Woman 4)

Additionally, both men and women identified that decisions and preparation for going to the hospital for delivery should be made early in the pregnancy.

When you are pregnant, you need to prepare yourself and keep some money, you never know when the day will come. If you have your money prepared, you can even pay for surgery if required. (FGD, Woman 6)

### Theme 2: Preference for hospitals over traditional birth attendants for delivery

Women and men expressed a strong desire to have the mother deliver in a health facility over using TBAs and having a home birth. This was related to the greater quality of care they received health facilities, recognizing the ability of health facilities to keep women and infants safe, particularly if a complication arises. This arose both from previous experience delivering in one or both of the facilities as well as advice they had received from friends and family who had experience in different facilities.

In the hospital, I’m reassured about my wellbeing and that of my coming baby by the fact that medical personnel are experienced on the matters concerning delivery. At home, people assisting my delivery have neither knowledge nor experience on these matters. In case of any complications they may start fumbling and trying things at the expense of our lives. In the hospital, I’m sure nurses are present and in case they fail, they can call a doctor. I think there are even some diseases that could be transmitted during the process of labor. If in the hospital, I’m sure doctors understand preventative measures and they either do or tell me what to do. For example, I was told to press on belly to prevent further bleeding. (FGD, Woman 1)

Even within the hospitals, there was a preference for the use of private hospitals compared to public hospitals, mostly for the better quality of care received at private hospitals. While private hospitals cost more money, they often provided better care and had received better care from nurses.

My wife said that the doctors in the [private hospital] were entirely dedicated to providing high quality care for her with the state of the art facilities and a wide range of support services. But this service was lacking at the public hospital where nurses were not trained and very rude. They don’t care and they look at you as if you don’t need their services. (SSI, Man 3)

A father explained from his perspective of a motorcycle driver who has helped other women go to hospitals to deliver as well, explaining that even the service at reception is noticeably different.

I work as a motorcycle driver and I normally carry pregnant women to hospitals when they go to deliver…based on my experience, there is great difference on the welcoming care of private hospitals as compared to that of public hospitals. When you arrive at private hospitals nurses must greet you and you are given a cordial welcome. The nurse helped us to bring down the woman whose clothes were dirty due to the fluid that was coming out of her body. Unlike in public hospitals, she could have received abusive words and possibly slapped because of wearing dirty clothes as a result of the fluids. (FGD, Man 5)

Despite the desire to deliver at a private hospital, women and men also expressed a challenge with accessing quality health facilities near their homes for birth and thus, still relying on the use of public health facilities or TBAs. Participants also expressed financial challenges impacting their decision about where women deliver, suggesting that although the service was notably better in private hospitals, the costs were too high, resulting in a decision to delivery elsewhere.

In private hospitals, the service is really great but the cost is too high. For example, for those who go to deliver at [a private] hospital in Dar es Salaam, the service is superb but not everyone can afford it. (FGD, Woman 4)

### Theme 3: Who is involved in decision making around delivery location

Among the men, there was limited consensus about who was responsible for choosing where their wife delivery, some saying they were responsible for the decision alone, some mentioned it was a joint decision, and finally some said the women decided. Men also mentioned other individuals that may influence where a wife delivers, such as parents, neighbours and nurses.

Always a man is the president of the house who has a final decision on the delivering place of a pregnant woman. (FGD, Man 10)

She chose the place herself…she said that delivery might come with complications so it is better if she delivers from a place where she can get the help she needs just in case she encounters problems. (SSI, Man 4).

Both father and wife might share views on the delivering place…availability of services, accessibility and affordability of costs of services and transportation to reach the facility…are the motives behind the selection. (SSI, Man 6)

In case you live near the women’s parents, they will always want their daughter to deliver at their living premise…[but] I am always not ready to allow my wife to deliver at her parents’ home. (FGD, Man 1)

Neighbours may also have an impact on the decision [about where to deliver] especially in the absence of a husband. However, in most cases, men have final ruling on where a pregnant woman goes for delivery. (FGD, Man 7)

It depends with the attendance of pregnant women to clinics. Through examining the position of a baby in the womb, nurses will advise both about the safe place for giving birth… (FGD, Man 9)

Alternative to the mixed reporting from men, women reported that they made the decision themselves about where to deliver. However, women did report that other individuals played a role in her decision about where she delivered including healthcare providers, their own mothers, her husband, or having it a joint decision between herself and her husband.

I made the choice myself. I love this hospital because they give me good services. [If I were to make the choice again] I would still choose the same hospital. I chose this place because I love the place. (SSI, Woman 6)

From my experience, it’s me who has the final say on where I’ll give birth from. I prefer a center where there is a doctor on duty. During the last ANC visit, the attendant usually reminds me that my due date is near and encourages me not to forget to go to the nearest health facility when labour begin. From there, it’s my responsibility to make sure I comply. (FGD, Woman 11)

My mother [chose this place] because they take very good care of the clients. (SSI, Woman)

I decided [to give birth here] with my husband when we considered the nearness of the place and also our income. (FGD, Woman 12).

### Theme 4: Who attends the delivery

In relation to who attends the delivery and goes to the hospital, men expressed that they sometimes were not able to be involved in the delivery process, despite desiring to be. Men are not typically allowed to be present during delivery and are often required to stay outside in the waiting area or are told to go home and return later to pick up his wife and child. While some men expressed a desire to attend delivery, this was not universal.

I think that would be awesome [to be present when she is giving birth] because if the husband is present, she gets more energetic and even you can play the role of reassuring her by telling her that this is both our babies and it would be really great…even in the hospitals, I think if husbands are allowed in, it would be better for our wives. (SSI, Man 2)

I do not think it is appropriate for me to be nearer or directly observe my wife when delivering (FGD, Man 8)

On the other hand, women expressed a desire to have the father involved during the delivery process, mostly so that men could be aware of the birthing process. Yet, some women also acknowledged that despite their desire to have the father involved, the men were not interested, not able, or not ready to be involved.

I’d love for the father to escort me and if necessary, for him to be in the labour room to witness the ordeal that I’m going through. Yes, he should see that’s it. (SSI, Woman 5)

In real life, men are very reluctant to participate in matters concerning pregnancy and delivery. Most of us are escorted by our neighbours and other relatives, but is really encouraging when your husband escorts you too. In my case, in all my previous deliveries, my husband didn’t escort me. I was hoping he would escort me in this last pregnancy. Some people tried to help me to persuade [my husband to come with me to the hospital] but his reply was that he had given me money so I should just hire a taxi. Some old women stood up against him and made sure he came with us. It really felt great to have him there, even though he stayed at the hospital entrance. (FGD, Woman 3)

… men are just difficult...We mothers go through a lot and after that they claim ownership of the children while it’s us mothers who do the job of bringing them to this world, I think they should participate fully. (FGD, Woman 12)

If a father was not available, women also expressed a desire for others to be involved in the delivery process and escorting them to the hospital, particularly women. Other women can offer encouragement, particularly mothers or other family members. Women expressed a desire to have a companion as the nurses were often busy and while encouraging, the additional support of a companion was desired, yet not available, during the delivery process.

My mother was there [at the hospital with me] but she was told to wait outside. [I would have preferred she was present] because she was encouraging me and giving some instructions on how to go about it. But she was given so little time. The nurses hold her to tell me what to do because I was afraid so she advised me and later left me with the nurses who were also encouraging me. (SSI, Woman 5)

## Discussion

Overall, the main theme of this research was accessing delivery health services. In particular, women and men expressed several factors that influenced when and where they accessed services as well as, who was involved in the delivery. Increasingly, women are delivering in health facilities and as a result, the need to understand when women are going to the hospital, where women are choosing to deliver, and who is making that decision and involved in the delivery is essential. Understanding the facilitators and barriers that encourage health facility delivery is necessary to provide respective maternal care and humanize the birth experience for women in Tanzania.

When decisions are made about going to the hospital, there are sometimes challenges faced that result in delayed treatment seeking, including financial or location-based, but some couples are recognizing the need to seek care earlier to prevent complications arising and improve quality of care received. This was also noted in a study by Mselle et al. [8] with Tanzanian women who developed obstetric fistula as an outcome of their labour whereby struggles in reaching a health facility were due to facilities being too far and lack of access to transport. Other studies have also identified barriers in rural Tanzania for women accessing maternal services at health facilities including long distances to health facilities [19], the availability of TBA closer to their homes [19], and disagreement with partners about when to leave for the health facility [20]. Despite a growing desire for women to give birth at health facilities, challenges remain when a decision has already been made. These challenges limit the ability for women to receive quality care and improve respectful maternal care as they often arrive late and occasionally with complications due to delays in seeking treatment.

Additionally, there was a significant preference on where women are delivering, with private hospitals being the preferred location, followed by public hospitals and at home with TBA due to better quality of care received, yet again depends on financial reason. Both men and women had concerns around using a TBA who may not be able to address serious complications that arise, and expressed that hospitals were more likely to provide the quality of care they were looking forward. This growing preference as identified in our study for hospital delivery is supported by a recent review [21], occurring in both rural and urban areas, yet maternal and newborn mortality rates continue to remain high. This has been attributed the fact that the increase in facility deliveries does not typically corresponded to an increase in number of staff and facility infrastructure in these facilities, resulted in low quality care and increased burden on the health system and healthcare workers [21]. This leads to overcrowding, lack of adequate infrastructure, and lack of equipment needed for birth, including beds and sheets [22]. Nevertheless, as evident in our study, the quality of care received in hospitals was perceived to be of higher quality and less risky than having a home birth with a TBA, suggesting an increase in recognition in benefits of delivery at health facility.

However, there was also an obvious divide in the desire for private versus public hospitals, with most participants preferring the care received in the private hospital, saying that the nurses were more respectful during the birthing process and that women were attended to promptly and provided with better care. This came from both personal experience as well as word-of-mouth experience of other women, including mothers, sisters, and friends. It is not sufficient that women are able to access any health facility, but it is essential that quality services are provided at all health facilities. Using a hospital and community survey, Straneo et al. [23] found that rural Tanzanian women were disadvantaged in both access and quality of delivery care. Currently, both women and men have noticed a significant difference in the care provided in private and public facilities which is a significant issue that needs to be addressed in order to truly provide respectful birth services and caring to women across Tanzania. If women experience inhumane care at public facilities, they may choose to deliver at home instead, despite the risks, if they cannot afford private care. Most previous studies have compared between home delivery and health facilities [24–26], yet our study suggests that even within health facilities there is a factors that exist the influences which type of health facility women are delivering in.

Finally, there is mixed evidence on who is involved in decision making around delivery location, depending on whether one is asking women or men. A previous study in Tanzania noted that having both partners agree that delivering at a health facility was important as well as a women’s preference for hospital delivery increased the chances of actually delivering at a health facility [14]. Men were quite mixed when reporting who was the primary decision maker about the delivery location, whereas the women primarily reported that they were the ones who decided where they should deliver. Men in this study also expressed a desire to have a woman deliver at the hospital compared to using a TBA who may not be able to address and serious complications that arise. This differed from the findings by Mselle et al. [8] who found that men preferred home deliveries and were the ones predominately making decisions around where a women delivered. Other studies in Tanzania have found that women’s autonomy around seeking maternal care continues to be influenced by family members, particularly in rural areas [27, 28]. Our findings are unique and provide some positive indications that cultural norms may be shifting towards more equality in decision making around delivery care as well as preference for hospital deliveries in some areas of Tanzania. As men become more aware of the importance of seeking early care and become more involved in antenatal and delivery process, it can increase shared decision making, knowledge, and accompaniment to health facilities for delivery [29]. These cultural shifts are positive and should be capitalized on to further encourage the involvement of men in maternal health care to be empowering for both men and women in humanizing the birth process [30].

Despite the differences in who selected the delivery location, both groups expressed a preference for more male involvement during the delivery. As indicated by the men in this study, some of them were interested in being with their partner while she was giving birth, yet their ability to be involved continued to be limited. Men continued to be told to wait in the reception area while the women went through labour, despite desire expressed by both women and men to have them involved in the process. In order for men to be increasingly involved in maternal health care, traditional gender norms and institutional barriers need to be removed [31]. The most recent World Health Organization recommendation on intrapartum care recognizes that a key component of a positive childbirth experience is providing respectful care, including the physical and emotional support from a companion of their choice during delivery [12]. However, the concept of the labour room as a space for women only continues to dominate the discourse in several low resource countries [30]. Furthermore, the facility’s physical infrastructure limits the ability for men to present at the bedside during labour as many hospitals are limited in physical space and overcrowding without having additional bodies in the labour room [22]. It should be noted that not all men expressed a desire to be involved in the birth process, with some men indicating that they are not comfortable with being present while their wife is delivering. In humanizing the birth process, it is essential that this is also taken into account to ensure that the rights and desires of both the wife and father are respected.

This study had many strengths including the inclusion of both men and women to understand the perceptions how decisions are made around where a woman gives birth in Tanzania, understanding that family and contextual factors are important. However, a limitation of this research is that participants were recruited from a postnatal clinic and had recently delivered at a hospital, and thus might reflect a bias towards hospital deliveries compared to TBA practices and may have missed some women and men who lost a child, potentially during delivery or shortly after, which may influence their perceptions of facility and/or home births. We also did not recruit primiparas mothers and thus may have missed capturing some of the differences that influence women in making their decision location for their first birth. However, attempts were made to gain a diverse sample of participants within our targeted population and through the interviews, it was clear that women and men had different experiences with deliveries, both at home and at hospitals, and thus were able to reflect on the difference between them. Another limitation is that the analysis of the FGDs and SSIs were completed in English from translated transcripts. However, the transcripts were verified by co-authors fluent in Kiswahili to ensure adequate translations and all codes and themes were discussed amongst the co-authors who were able to review the original transcripts.

## Conclusion

This study shows that increasingly men and women are desiring for women to delivery at health facilities yet there is still a need to improve respective maternal care and humanize the birth process in Tanzania. Furthermore, this study shows evidence that both men and women desire for greater male involvement in the birth process in Tanzania, yet systematic challenges limit the ability for this to occur. To fully humanize the birth care in Tanzania, greater emphasis needs to be placed on including men and other family members during the birth process, improving the quality of care received in public hospitals, and reducing the barriers to accessing health facilities for delivery care.

## Abbreviations

FGD: Focus group discussions
SSI: Semi-structured interviews
TBA: Traditional birth attendant
